# Novel biomarkers of hidden inflammation and implantation

**DOI:** 10.1186/1878-5085-5-S1-A116

**Published:** 2014-02-11

**Authors:** Julia Kzhyshkowska, Ilja Ovsiy, Alexei Gratchev

**Affiliations:** 1 Medical Faculty Mannheim, Ruprecht-Karls University of Heidelberg, Theodor-Kutzer Ufer 1-3, 68167, Mannheim, Germany

## 

Successful implantation of dental implants depends not only on the implant material, but is also significantly affected by the immunologic status of the organism. The most problematic here is chronic low grade inflammation which increases with the age (i.e., immunaging). This type of inflammation differs from classical acute inflammation by the absence of clinical symptoms and specific activation of monocytes in the circulation and tissue macrophages. Highly specific and sensitive biomarkers for detection of this inflammatory condition are urgently needed. In our laboratory, we have identified several biomarkers that are expressed by macrophages differentiated in the microenvironment typical for chronic low grade inflammation. These markers include receptors Stabilin-1 and IL17RB, and transcription factor FoxQ1. Stabilin-1 is a multifunctional macrophage receptor responsible for scavenging of unwanted self products and for sorting of newly synthesized ligands and facilitating their secretion. These functions of Stabilin-1 indicate that it is not only a marker, but also actively involved in the regulation of inflammatory reactions. IL17RB is a receptor for IL17E – the sole member of IL17 family that is not involved in regulation of type 1 inflammation, but rather mediating low grade type 2 inflammation. FoxQ1 is a transcription factor of forkhead family. The entire family is actively involved in regulation of innate and adaptive immune reactions including the development of regulatory T-cell phenotype. Our data indicate that FoxQ1 is typical for macrophages showing increased adhesion and migration capabilities, making them active players in low grade inflammation in tissues. All above mentioned biomarkers are expressed by monocytes/macrophages upon stimulation with IL-4 or in combination with TGFβ and/or IFNγ- cytokines playing important role in chronic low grade inflammation. Association of Stabilin-1, FoxQ1 and IL17RB with chronic low grade inflammation makes them not only viable personalized diagnostic markers, but also suggests their usefulness as targets for development of novel personalized therapeutic options. We propose to use novel biomarkers of chronic inflammation to predict individual reactions of patients to implants and as a reference for development of immunostabilizing therapies to prevent implant failure.

